# A dynamic residential community-based quarantine strategy: China’s experience in fighting COVID-19

**DOI:** 10.1017/ice.2020.172

**Published:** 2020-04-23

**Authors:** Yan Guo, Yiran Li, Aliza Monroe-Wise, Sai-Ching Jim Yeung, Yixiang Huang

**Affiliations:** 1 Sun Yat-sen Global Health Institute, Sun Yat-sen University, Guangzhou, China; 2 Department of Medical Statistics, School of Public Health, Sun Yat-sen University, Guangzhou, China; 3 Department of Global Health, University of Washington, Seattle, Washington, United States; 4 Department of Emergency Medicine, The University of Texas MD Anderson Cancer Center, Houston, Texas, United States; 5 Department of Health Policy and Management, School of Public Health, Sun Yat-sen University, Guangzhou, China

*To the Editor*—As the global COVID-19 pandemic progresses, many countries face major public health emergencies. The number of active COVID-19 cases worldwide is >1.69 million as of April 12, 2020.^1^ With rapidly increasing new cases, healthcare systems are at the brink of collapse in some regions. To reduce the burden on health systems, public health strategies should be adopted to control the source of infection, to cut off transmission routes, and to protect vulnerable populations. One strategy that was effective in China in controlling the COVID-19 epidemic was the successful implementation of a nationwide, community-based, dynamic quarantine strategy.^2^ From Late January to March 18, 2020, the main purpose was to prevent COVID-19 from spreading in China. Afterward, the focus turned to the prevention of imported cases.

At the beginning of China’s quarantine, residents were required to stay home. When necessary, they were required to use an electronic pass system with traceable personal information to gain entry to residential areas, work places, and public transportation, and body temperature was screened by thermal scanning at the entrances.^3^ Once new cases were identified, health professionals and volunteers followed-up, treated, and isolated the patient and those in close contact. From prior experience with SARS, most Chinese citizens understood the importance of the quarantine and were invested in its success. As the epidemic came under better control, community-level management was strengthened and upgraded. It also became more dynamic, allowing movement and return to work to minimize the effects on people’s lives and businesses while still monitoring movements and health status.^4^

Experience can be drawn from China’s quarantine strategy. First, the quarantine strategy was strictly implemented nationwide at a community level.^5^ Strong governmental support is required to strengthen the community, and training is needed to foster policy implementation. Second, the quarantine strategy was dynamic and was adjustable based on the evolving situations, from stay-home quarantine to movement with updated health monitoring. Third, a large team of professional and technical support traced, identified, treated, and isolated patients and their close contacts. These strategies ensured early diagnosis and treatment, thus bringing the COVID-19 pandemic under control in China.

As the COVID-19 pandemic continues to take a massive toll on the healthcare systems and economies of countries worldwide, China’s experience in its fight against this novel virus can be of great value to other countries. A practical attitude of learning by doing and responsiveness among government officials and the general population must be fostered.
